# Supplementary material for: Outcome of Newborns with Confirmed or Possible SARS-CoV-2 Vertical Infection—A Scoping Review
Source: Diagnostics (Basel). 2023 Jan 9;13(2):245. doi: 10.3390/diagnostics13020245 (PMC9858608; doi:10.3390/diagnostics13020245)
Supplement: Supplementary file 1 [file diagnostics-13-00245-s001.zip › supplemental S1.pdf]

Requirement for correct classification of vertical transmission of SARS-CoV-2 in livebirths [1], [2]

Table S1. SARS-CoV-2 intrauterine exposure could be classified as confirmed and possible using the WHO criteria, or confirmed, probable or possible according to Shah's criteria. each categorization is color coded. The probable and possible cases according to Shah are grouped under the same category: possible. **"+"-** positive test; **ND –** not done. We will further give an example to better understand how to read the table. If neonate X has a positive amniotic fluid at birth, AND **1.** there are no additional test, he could be diagnosed as confirmed IUE according to Shah
2. there is a positive result from neonatal blood in the 24-48h timeframe, he could be diagnosed as confirmed IUE according to the WHO criteria **3.** there is a positive result from neonatal nasopharyngeal swab in the 24-48h timeframe, he could be diagnosed as possible IUE according to the WHO criteria

		RT-PCR from sterile sample								placental tissue analysis		RT-PCR from non-sterile		Serology (IgM or IgA)
		amniotic fluid		neonatal blood		lower respiratory tract		cord blood				upper respiratory tract samples		
one of the criteria	0-24h	+	+	+	+*	+	ND	+	+	+*	+*	+	+**	+
		+	ND	+	ND	+	ND	+	ND	+*	ND	+	+	+
and														
one of the criteria	24-48h	ND	ND	+	+	ND	+	ND	ND	ND	ND	+	+*	+**
		ND	ND	+	ND	+	ND	+	ND	ND	ND	ND	+	+*

Intra-uterine exposure		confirmed according to WHO<br>*only RT-PCR and ISH to be used
		confirmed according to Shah's criteria<br>* in the first 12 h of life
		possible according to WHO<br>*RT-PCR, ISH, IHC or microscopy<br>**24h-7days
		probable according to Shah's criteria<br>*either swab from maternal side, or placental tissue examination<br>**and nasopharyngeal swab at birth

Intrapartum exposure		confirmed according to WHO (as long as all tests performed before 24 hours of age were negative)<br>* 7-14 days that is corroborated by second sample obtained within 10 days of the first positive test
		confirmed according to Shah<br>* nasopharyngeal swab at birth
		possible according to WHO (as long as there are no tests performed before 24 hours of age)<br>*providing that there is a second positive PCR from non-sterile sample between 48h-7days

Requirement for correct classification of vertical transmission of SARS-CoV-2 in stillbirths

Table S2. SARS-CoV-2 intrauterine exposure could be classified as confirmed and possible using the WHO criteria, or confirmed, probable or possible according to Shah's criteria. each categorization is color coded. The probable and possible cases according to Shah are grouped under the same category: possible. **"+"-** positive test; **ND** – not done.

RT-PCR from				analysis placental tissue	
fetal tissue		amniotic fluid			
+*	+*	ND	+	ND	+**
+*	+*	ND	ND	+*	+**

	confirmed according to WHO<br>* ISH can be used
	confirmed according to Shah<br>* RT-PCR, microscopy, or viral growth in culture
	possible according to WHO (providing that fetal tissue was not tested; one criteria is enough)<br>*IHC or microscopy or fetal swab RT-PCR<br>** RT-PCR, ISH, IHC or microscopy) or placental swab RT-PCR
	possible according to Shah's criteria<br>*RT-PCR swab from fetal surface<br>**RT-PCR swab from fetal side

Reference

1. Shah, P.S.; Diambomba, Y.; Acharya, G.; Morris, S.K.; Bitnun, A. Classification system and case definition for SARS-CoV-2 infection in pregnant women, fetuses, and neonates. Acta Obstet. et Gynecol. Scand. 2020, 99, 565–568. https://doi.org/10.1111/aogs.13870.
2. Definition and categorization of the timing of mother-to-child transmission of SARS-CoV-2. Available online: https://www.who.int/publications-detail-redirect/WHO-2019-nCoV-mother-to-child-transmission-2021.1 (accessed on 3 November 2022).
