# Supplementary material for: Outcome of Newborns with Confirmed or Possible SARS-CoV-2 Vertical Infection—A Scoping Review
Source: Diagnostics (Basel). 2023 Jan 9;13(2):245. doi: 10.3390/diagnostics13020245 (PMC9858608; doi:10.3390/diagnostics13020245)
Supplement: Supplementary file 1 [file diagnostics-13-00245-s001.zip › supplemental S2.pdf]

Quality Assessment and Summary of Key Characteristics of Included Studies

Table S1. Case Reports/Case Series Studies assessed using the Murad Tool Scoring Criteria [1]. The study is classified as "good quality" – score 4, "fair quality" – score 3 and "poor quality" score between 1-2

Author	Country	Number of eligible newborns	Quality Assessment Score
**Abadía-Cuchí *et al* [2]**	Spain	1	2
**Alzamora MC *et al* [3]**	Peru	1	4
**Babal *et al* [4]**	Slovakia	1	2
**Boncompagni *et al*[5]**	Italy	1	3
**Buonsenso D *et al*[6]**	Italy	1	3
**Carvalho *et al*[7]**	Brazil	2	4
**Choobdar *et al*[8]**	Iran	1	3
**Correia *et al*[9]**	Portugal	1	2
**Disse *et al*[10]**	Germany	2	3
**Dong *et al*[11]**	China	1	4
**Enache *et al*[12]**	Romania	1	3
**Ergon *et al*[13]**	Turkey	1	3
**Facchetti *et al*[14]**	Italy	1	2
**Farhadi *et al*[15]**	Iran	1	4
**Favre *et al*[16]**	Switzerland	1	3
**Fitzgerald *et al*[17]**	Ireland	5	3
**Fusco *et al*[18]**	Brazil	3	4
**Gant *et al*[19]**	USA	1	4
**Gioia *et al*[20]**	Italy	1	2
**Karade *et al*[21]**	India	1	3
**Kato *et al*[22]**	Japan	1	3
**Kirtsman *et al*[23]**	Canada	1	3
**Kohorn *et al*[24]**	USA	1	3
**Lesieur *et al*[25]**	France	1	3
**Lorenz *N et al*[26]**	Germany	1	3
**Marinho *et al*[27]**	Brazil	1	4
**Marton *et al*[28]**	UK	1	2
**Marzollo *et al*[29]**	Italy	1	3
**Metodiev *et al*[30]**	Bulgaria	1	3

**NG David** ***et al*** **[31]**	Malaysia	1	3
**Parsa** ***et al*** **[32]**	Iran	1	4
**Patanè** ***et al*** **[33]**	Italy	2	4
**Popescu** ***et al*** **[34]**	Romania	1	2
**Pulinx** ***et al*** **[35]**	Belgium	2	2
**Reagan-Steiner** ***et al*** **[36]**	USA	1	3
**Rebello** ***et al*** **[37]**	Brazil	1	3
**Richtmann** ***et al*** **[38]**	Brazil	1	4
**Rodrigues** ***et al*** **[39]**	Portugal	1	2
**Schueda Stonoga** ***et al*** **[40]**	Brazil	1	2
**Sessa** ***et al*** **[41]**	Italy	2	3
**Shaiba** ***et al*** **[42]**	Saudi-Arabia	1	2
**Shen** ***et al*** **[43]**	USA	1	2
**Sukhikh** ***et al*** **[44]**	Russia	1	3
**Vivanti** ***et al*** **[45]**	France	5	2
**Vivanti** ***et al*** **[46]**	France	1	4
**Zaigham** ***et al*** **[47]**	Sweden	9	3
**Zamaniyan** ***et al*** **[48]**	Iran	1	4
**Zhang** ***et*** **[49]**	USA	1	2
**Zinserling** ***et al*** **[50]**	Germany	1	4

Table S2. Scoring Criteria for the Murad Tool

**Selection**	• Were the cases presented from a particular study period?
**Ascertainment**	• Were the cases positive for COVID-19 according to the accepted criteria?<br>• Was the neonates' clinical status reported?
**Causality**	• Have alternative causes for the presence of symptoms besides COVID-19 infection been ruled out?<br>• Was the duration of the follow-up sufficient for outcomes to occur, such as neonatal discharge or death?
**Reporting**	• Was the case described sufficiently for other researchers to replicate the study or for practitioners to draw inferences about their own practice?

Table S3. Studies were assessed based on Newcastle-Ottawa Scale. A study with a score of 6 or more is classified as high quality, 4-5 as medium quality, and less than 4 as low quality, using the Newcastle-Ottawa Scale [54].

Study	**Country**	**Cohort size**	**Number of cases with confirmed**	**Quality Assessment Score**

			vertical transmission	
Borges et al [51]	Portugal	527	1	6
Fenizia et al [52]	Italy	31	1	5
Garcia-Ruiz et al [53]	Turkey	43	1	5

Table S4. Scoring Criteria for the Newcastle-Ottawa Scale

**Selection**	• Were the cases presented representative of the average in the target population?<br>• Were there cases with negative COVID-19 test?<br>• Were the cases taken from medical records?<br>• Was the subject of vertical transmissions followed in the study from de beginning?
**Comparability**	• Were the cases of interest compared to other groups based on the study design or analysis?
**Outcome**	• Was there sufficient clinical data on the neonates?<br>• Were the confirmed cases followed up long enough for outcome to occur?<br>• Was there a complete follow-up of the confirmed cases?

Reference

1. Murad, M.H.; Sultan, S.; Haffar, S.; Bazerbachi, F. Methodological quality and synthesis of case series and case reports. *BMJ Evid. Based Med*. **2018**, 23, 60–63. https://doi.org/10.1136/bmjebm-2017-110853.
2. Abadía-Cuchí, N.; Ruiz-Martínez, S.; Fabre, M.; Mateo, P.; Remacha Sienes, M.; Ventura Faci, P.; Bueno Sancho, J. et al. SARS-CoV-2 congenital infection and pre-eclampsia-like syndrome in dichorionic twins: A case report and review of the literature. *Int. J. Gynaecol. Obstet. Off. Organ Int. Fed. Gynaecol. Obstet*. **2021**. 154(2), 370–372. https://doi: 10.1002/ijgo.13749.
3. Alzamora, M.C.; Paredes, T.; Caceres, D.; Webb, C.M.; Valdez, L.M.; La Rosa, M. Severe COVID-19 during Pregnancy and Possible Vertical Transmission. *Am. J. Perinatol*. **2020,** 37, 861–865. https://doi.org/10.1055/s-0040-1710050.
4. Babal, P.; Krivosikova, L.; Sarvaicova, L.; Deckov, I.; Szemes, T.; Sedlackova, T.; Palkovic, M.; Kalinakova, A.; Janega, P. Intrau-terine Fetal Demise After Uncomplicated COVID-19: What Can We Learn from the Case?. *Viruses*. **2021**. 13(12), 2545. doi: 10.3390/v13122545
5. Boncompagni, A.; De Agostini, M.; Lugli, L.; Ternelli, G.; Colonna, V.; Biagioni, E.; Bonasoni, M.P.; Salviato, T.; Gabrielli, L.; Falconi, M.; et al. Unexpected Vertical Transmission of SARS-CoV-2: Discordant Clinical Course and Transmission from Mother to Newborn. *Microorganisms.* **2022**. 10, 1718. https://doi:10.3390/microorganisms10091718
6. Buonsenso, D.; Costa, S.; Sanguinetti, M.; Cattani, P.; Posteraro, B.; Marchetti, S.; Carducci, B.; Lanzone, A.; Tamburrini, E.; Vento, G. Neonatal Late Onset Infection with Severe Acute Respiratory Syndrome Coronavirus 2. *Am. J. Perinatol*. **2020**. 37(8), 869–872. https://doi: 10.1055/s-0040-1710541.
7. Ferreira, M.D.F.C.; Pavon, J.A.R.; Napoleão, A.C.B.; Figueiredo, G.M.D.P.; Florêncio, P.C.B.; Arantes, R.B.d.S.; Rizzo, P.S.; Carmo, M.A.M.V.; Nakazato, L.; Dutra, V.; et al. Clinical and genomic data of sars-cov-2 detected in maternal–fetal interface during the first wave of infection in Brazil. **Microbes Infect.** *2022*, 24, 104949. https://doi.org/10.1016/j.micinf.2022.104949.

8. Choobdar, F.A.; Ghassemzadeh, M.; Attarian, M.; Abbariki, E.; Nateghian, A.; Ghanbari, B.; Hamzehi, S.S. et al. Transplacental Transmission of SARS-CoV-2 Infection: A Case Report from Iran. *Arch. Pedi-atr. Infect. Dis*. **2021**. 9(2), 3572. https://doi: 10.5812/pedinfect.108582
9. Correia, C.R.; Marçal, M.; Vieira, F.; Santos, E.; Novais, C.; Maria, A.T.; Malveiro, D.; Prior, A.R.; Aguiar, M.; Salazar, A.; et al. Congenital SARS-CoV-2 Infection in a Neonate With Severe Acute Respiratory Syndrome. *Pediatr. Infect. Dis. J*. **2020**, 39, e439–e443. https://doi.org/10.1097/INF.0000000000002941
10. Disse, S.C.; Manuylova, T.; Adam, K.; Lechler, A.; Zant, R.; Klingel, K.; Aepinus, C.; Finkenzeller, T.; Wellmann, S.; Schneble, F. COVID-19 in 28-Week Triplets Caused by Intrauterine Transmission of SARS-CoV-2—Case Report. *Front. Pediatr*. **2021**, 9, 812057. https://doi: 10.3389/fped.2021.812057
11. Dong, L.; Tian, J.; He, S.; Zhu, C.; Wang, J.; Liu, C.; Yang, J. Possible Vertical Transmission of SARS-CoV-2 From an Infected Moth-er to Her Newborn. *JAMA*. **2020**. 323(18), 1846–1848. https://doi: 10.1001/jama.2020.4621.
12. Enache, A.; Ciocan V.; Muresan C.O.; Cut, T.G.; Novacescu D. et al. Postmortem Documentation of SARS-CoV-2 in Utero and Postpartum Transmission, through Amniotic Fluid, Placental, and Pulmonary Tissue RT-PCR. *Appl. Sci***. 2021**. 11(20), 9505 .doi: 10.3390/app11209505
13. Yangin Ergon E, Akbay S, Aytemiz G, Avcı Çelik EC, Çalıskan Polat A, Umit Z, Paytoncu S.., "A novel case of neonatal acute respiratory distress syndrome with SARS-CoV-2 infection: potential perinatal transmission," Arch. Argent. Pediatr., vol. 119, no. 5, pp. e531–e535, Oct. 2021, doi: 10.5546/aap.2021.eng.e531.
14. Facchetti, F.; Bugatti, M.; Drera, E.; Tripodo, C.; Sartori, E.; Cancila, V.; Papaccio, M.; Castellani, R.; Casola, S.; Boniotti, M.B.; et al. SARS-CoV2 vertical transmission with adverse effects on the newborn revealed through integrated immunohistochemical, electron microscopy and molecular analyses of Placenta. *Ebiomedicine* **2020,** 59, 102951. https://doi:10.1016/j.ebiom.2020.102951
15. Farhadi, R.; Mehrpisheh, S.; Ghaffari, V.; Haghshenas, M.; Ebadi, A. Clinical course, radiological findings and late outcome in preterm infant with suspected vertical transmission born to a mother with severe COVID-19 pneumonia: A case report. *J. Med. Case Rep*. **2021**, 15, 213. https://doi:10.1186/s13256-021-02835-0.
16. Favre, G.; Mazzetti, S.; Gengler, C.; Bertelli, C.; Schneider, J.; Laubscher, B.; Capoccia, R.; Pakniyat, F.; Ben Jazia, I.; Eggel-Hort, B.; et al. Decreased Fetal Movements: A Sign of Placental SARS-CoV-2 Infection with Perinatal Brain Injury. *Viruses* **2021**, 13, 2517. https://doi:10.3390/v13122517.
17. Fitzgerald, B.; O'Donoghue, K.; McEntagart, N.; Gillan, J.E.; Kelehan, P.; O'Leary, J.; Downey, P.; Dean, J.; De Gascun, C.F.; Fetal Deaths in Ireland Due to SARS-CoV-2 Placentitis Caused by SARS-CoV-2 Alpha. *Arch. Pathol. Lab. Med*. **2022**. 146(5),529–537. doi: 10.5858/arpa.2021-0586-SA.
18. Fusco, M.A.; Mantini, V.; de Souza Salmont Júnior, J.; de Carvalho Gomes, R.G.; de Oliveira Lima, A.R.; de Oliveira Clarim, H.L.; Ferreira, E.C.; de Abreu Almeida, S.S.; Salmont, C.G.; de Sousa Rizzo-Valente, V.; et al. Assessment of SARS-CoV-2 Vertical Transmission through Nested RT-PCR Testing of Neonatal Samples: Three Case Reports. *J. Pediatr. Perinatol. Child Health* **2022**, 6, 370–376
19. Gant,T. F.; Villegas, T. P.; Summerall-Smith, J.; Watkins, B. Intrauterine fetal demise as a result of maternal COVID-19 infection in the third trimester of pregnancy: A case report. *Int. J. Surg. Case Rep*. **2022.** 98,107492, doi: 10.1016/j.ijscr.2022.107492.
20. di Gioia, C.; Zullo, F.; Bruno Vecchio, R.C.; Pajno, C.; Perrone, G.; Galoppi, P.; Pecorini, F.; Di Mascio, D.; Carletti, R.; Prezioso, C. et al. Stillbirth and fetal capillary infection by SARS-CoV-2. *Am. J. Obstet. Gynecol*. **2022**. MFM. 4(1),100523. doi: 10.1016/j.ajogmf.2021.100523.
21. Karade, S., Vishal, A. K.; Sen, S.; Bewal, N.; Gupta, R. M. Probable vertical transmission of severe acute respiratory syn-drome coronavirus 2 infection from mother to neonate. *Med. J. Armed Forces India*. **2021**. 77(2) S490–S493. https://doi: 10.1016/j.mjafi.2020.11.026
22. Kato, M.; Yamaguchi, K.; Maegawa, Y.; Komine-Aizawa, S.; Kondo, E.; Ikeda, T. Intrauterine fetal death during COVID-19 pregnancy: Typical fetal heart rate changes, coagulopathy, and placentitis. *J. Obstet. Gynaecol. Res*. **2022**. vol. 48(7), 1978–1982., doi: 10.1111/jog.15302
23. Kirtsman, M.; Diambomba, Y.; Poutanen, S.M.; Malinowski, A.K.; Vlachodimitropoulou, E.; Parks, W.T.; Erdman, L.; Morris, S.K.; Shah, P.S. Probable congenital SARS-CoV-2 infection in a neonate born to a woman with active SARS-CoV-2 infection. *Can. Med. Assoc. J*. **2020**, 192, E647–E650. https://doi:10.1503/cmaj.200821.
24. Von Kohorn, I.; Stein, S.R.; Shikani, B.T.; Ramos-Benitez, M.J.; Vannella, K.M.; Hewitt, S.M.; E Kleiner, D.; Alejo, J.C.; Burbelo, P.; I Cohen, J.; et al. In Utero Severe Acute Respiratory Syndrome Coronavirus 2 Infection. *J. Pediatr. Infect. Dis. Soc*. **2020**, 9, 769–771. https://doi.org/10.1093/jpids/piaa127

25. Lesieur, E.; Torrents, J.; Fina, F.; Zandotti, C.; Blanc, J.; Collardeau-Frachon, S.; Gazin, C.; Sirgant, D.; Mezouar, S.; Otmani Idrissi, M.; Lepidi, H. Congenital Infection of Severe Acute Respiratory Syndrome Coronavirus 2 With Intrauterine Fetal Death: A Clinicopathological Study With Molecular Analysis. *Clin. Infect. Dis. Off. Publ. Infect. Dis. Soc. Am*. **2022**. 75(1), e1092–e1100. doi: 10.1093/cid/ciab840
26. Lorenz, N.; Treptow, A.; Schmidt, S.; Hofmann, R.; Raumer-Engler, M.; Heubner, G.; Gröber, K. Neonatal Early-Onset Infection With SARS-CoV-2 in a Newborn Presenting With Encephalitic Symptoms. *Pediatr. Infect. Dis. J.* **2020**, 39, e212. https://doi.org/10.1097/INF.0000000000002735
27. Marinho, P.S.; da Cunha, A.J.L.A.; Chimelli, L.; Avvad-Portari, E.; Andreiuolo, F.D.M.; de Oliveira-Szejnfeld, P.S.; Mendes, M.A.; et al. Case Report: SARS-CoV-2 Mother-to-Child Transmission and Fetal Death Associated With Severe Placental Thromboem-bolism. *Front. Med*. **2021**. 8,677001. doi: 10.3389/fmed.2021.677001.
28. Marton, T.; Hargitai, B.; Hunter, K.; Pugh, M.; Murray, P. Massive Perivillous Fibrin Deposition and Chronic Histiocytic Intervillositis a Complication of SARS-CoV-2 Infection. *Pediatr. Dev. Pathol*. **2021** 24(5), 450–454, doi: 10.1177/10935266211020723.
29. Marzollo, R.; Aversa, S.; Prefumo, F.; Saccani, B.; Perez, C.R.; Sartori, E.; Motta, M. Possible Coronavirus Disease 2019 Pandemic and Pregnancy: Vertical Transmission Is Not Excluded. Pediatr. *Infect. Dis. J*. **2020**, 39, e261–e262. https://doi:10.1097/INF.0000000000002816.
30. Metodiev, D.; Ruseva, M.; Parvanov, D.; Ganeva, R.; Handzhiyska, M.; Vidolova, N.; Stamenov, G. Vertical Transmission of SARS-CoV-2 Infection and Miscarriage in the Second Trimester: Report of an Immunohistochemically Proven Case. *Clin. Pract*. **2022**. 12 (4), 579–590, https://doi: 10.3390/clinpract12040061.
31. Ng, D.C.; Chin, L.; Choo, P.P.L.; Paramasivam, U. COVID-19 in a premature infant. *BMJ Case Rep*. *2021*, 14, e243783. https://doi:10.1136/bcr-2021-243783Y.
32. Parsa, Y.; Shokri, N.; Jahedbozorgan, T.; Naeiji, Z.; Zadehmodares, S.; Moridi, A. Possible Vertical Transmission of COVID-19 to the Newborn; a Case Report. *Arch. Acad. Emerg. Med*. **2021.** 9(1):e5. https://doi: 10.22037/aaem.v9i1.923.
33. Patanè, L.; Cadamuro, M.; Massazza, G.; Pirola, S.; Stagnati, V.; Comerio, C.; Carnelli, M.; Arosio, M.; Callegaro, A.P.; Tebaldi, P., et al. Evidence of vertical transmission of SARS-CoV-2 and interstitial pneumonia in second-trimester twin stillbirth in asymp-tomatic woman. Case report and review of the literature. *Am. J. Obstet. Gynecol. MFM*. *2022*. 4(3), 100589, doi: 10.1016/j.ajogmf.2022.100589.
34. Popescu, D.E.; Cioca, A.; Muresan, C.; Navolan, D.; Gui, A.; Pop, O.; Marcovici, T.; Ilie, C.; Craina, M.; Boia, M. A. A Case of COVID-19 Pregnancy Complicated with Hydrops Fetalis and Intrauterine Death. *Med. Kaunas Lith*. **2021**. 57(7), 667 doi: 10.3390/medicina57070667.
35. Pulinx, B.; Kieffer, D.; Michiels, I.; Petermans, S.; Strybol, D.; Delvaux, S.; Baldewijns, M.; Raymaekers, M.; Cartuyvels, R.; Maurissen, W. Vertical transmission of SARS-CoV-2 infection and preterm birth. *Eur. J. Clin. Microbiol. Infect. Dis*. **2020**, 39, 2441–2445. https://doi.org/10.1007/s10096-020-03964-y.
36. Reagan-Steiner, S.; Bhatnagar, J.; Martines, R.B.; Milligan, N.S.; Gisondo, C.; Williams, F.B.; Lee, E.; Estetter, L.; Bullock, H.; Goldsmith, C.S.; et al. Detection of SARS-CoV-2 in Neonatal Autopsy Tissues and Placenta. *Emerg. Infect. Dis*. **2022,** 28, 510–517. https://doi:10.3201/eid2803.211735
37. Rebello, C.M.; Fascina, L.P.; Annicchino, G.; Pinho, J.R.R.; Yoshida, R.D.A.M.; Zacharias, R.S.B. Vertical transmission of SARS-CoV-2 from infected pregnant mother to the neonate detected by cord blood real-time polymerase chain reaction (RT-PCR). *Pediatr. Res*. **2020**, 89, 1592–1593. https://doi:10.1038/s41390-020-01193-9.
38. Richtmann, R.; Torloni, M.R.; Oyamada Otani, A.R.; Levi, J.E.; Crema Tobara, M.; de Almeida Silva, C.; Dias, L.; Miglioli-Galvão, L.; Martins Silva, P.; Macoto Kondo, M.; Fetal deaths in pregnancies with SARS-CoV-2 infection in Brazil: A case series. *Case Rep. Womens Health*. **2020**. 27, e00243. doi: 10.1016/j.crwh.2020.e00243.
39. Rodrigues, M. L.; Gasparinho, G.; Sepúlveda, F.; Matos, T. Signs suggestive of congenital SARS-CoV-2 infection with in-trauterine fetal death: *A case report. Eur. J. Obstet. Gynecol. Reprod. Biol.* **2021**. 256,508–509. doi: 10.1016/j.ejogrb.2020.11.042.
40. Stonoga, E.T.S.; Lanzoni, L.D.A.; Rebutini, P.Z.; de Oliveira, A.L.P.; Chiste, J.A.; Fugaça, C.A.; Prá, D.M.M.; Percicote, A.P.; Rossoni, A.; Nogueira, M.B.; et al. Intrauterine Transmission of SARS-CoV-2. *Emerg. Infect. Dis*. **2021**, 27, 638–641. https://doi.org/10.3201/eid2702.203824.
41. Sessa, R.; Masciullo, L.; Filardo, S.; Di Pietro, M.; Brandolino, G.; Brunelli, R.; Galoppi, P.; Terrin, G.; Viscardi, M.F.; Anastasi, E.; et al. SARS-CoV-2 vertical transmission in a twin-pregnant woman: A case report. *Int. J. Infect. Dis*. **2022**, 125, 192–194. https:// doi: 10.1016/j.ijid.2022.10.019.

42. Shaiba, L.A.; Hadid, A.; Altirkawi, K.A.; Bakheet, H.M.; Alherz, A.M.; Hussain, S.A.; Sobaih, B.H.; Alnemri, A.M.; Almaghrabi, R.; Ahmed, M.; et al. Case Report: Neonatal Multi-System Inflammatory Syndrome Associated With SARS-CoV-2 Exposure in Two Cases From Saudi Arabia. *Front. Pediatr*. **2021**, 9, 652857. https://doi:10.3389/fped.2021.652857.
43. Shen, W.-B.; Turan, S.; Wang, B.; Cojocaru, L.; Harman, C.; Logue, J.; Reece, E.A.; Frieman, M.B.; Yang, P. A SARS-CoV-2 Delta Variant Case Manifesting as Extensive Placental Infection and Fetal Transmission. *Gynecol. Obstet. Investig*. **2022**, 87, 165–172. https://doi.org/10.1159/000524905.
44. Sukhikh, G.; Petrova, U.; Prikhodko, A.; Starodubtseva, N.; Chingin, K.; Chen, H.; Bugrova, A.; Kononikhin, A.; Bourmenskaya, O.; Brzhozovskiy, A.; et al. Vertical Transmission of SARS-CoV-2 in Second Trimester Associated with Severe Neonatal Pathology. *Viruses* **2021**, 13, 447. https://doi:10.3390/v13030447
45. Vivanti, A.J.; Vauloup-Fellous, C.; Escourrou, G.; Rosenblatt, J.; Jouannic, J.-M.; Laurent-Bellue, A.; De Luca, D. Factors associated with SARS-CoV-2 transplacental transmission. *Am. J. Obstet. Gynecol*. **2022**, 227, 541–543.e11. https://doi.org/10.1016/j.ajog.2022.05.015.
46. Vivanti, A.J.; Vauloup-Fellous, C.; Prevot, S.; Zupan, V.; Suffee, C.; Do Cao, J.; Benachi, A.; De Luca, D. Transplacental transmission of SARS-CoV-2 infection. *Nat. Commun*. **2020**, 11, 3572. https://doi.org/10.1038/s41467-020-17436-6.
47. Zaigham, M.; Gisselsson, D.; Sand, A.; Wikström, A.; von Wowern, E.; Schwartz, D.A.; Iorizzo, L.; Nelander, M.; Blomberg, M.; Papadogiannakis, N.; et al. Clinical-pathological features in placentas of pregnancies with SARS-CoV -2 infection and adverse outcome: Case series with and without congenital transmission. *BJOG: Int. J. Obstet. Gynaecol*. **2022**, 129, 1361–1374. https://doi.org/10.1111/1471-0528.17132.
48. Zamaniyan, M.; Ebadi, A.; Aghajanpoor, S.; Rahmani, Z.; Haghshenas, M.; Azizi, S. Preterm delivery, maternal death, and vertical transmission in a pregnant woman with COVID-19 infection. *Prenat. Diagn*. **2020**, 40, 1759–1761. https://doi.org/10.1002/pd.5713
49. Zhang, P.; Salafia, C.; Heyman, T.; Salafia, C.; Lederman, S.; Dygulska, B. Detection of severe acute respiratory syndrome coronavirus 2 in placentas with pathology and vertical transmission. *Am. J. Obstet. Gynecol. MFM*. **2020**. 2(4),100197. https://doi: 10.1016/j.ajogmf.2020.100197.
50. Zinserling, V.A.; Bornstein, S.R.; Narkevich, T.A.; Sukhanova, Y.V.; Semenova, N.Y.; Vashukova, M.A.; Steenblock, C. Stillborn child with diffuse SARS-CoV-2 viral infection of multiple organs. Idcases 2021, 26, e01328. https://doi.org/10.1016/j.idcr.2021.e01328
51. Borges Charepe, N.; Queirós, A.; Alves, M.J.; Serrano, F.; Ferreira, C.; Gamito, M.; Smet, C.; Silva, V.; Féria, B.; Laranjo, M.; Martins, I. One Year of COVID-19 in Pregnancy: A National Wide Collaborative Study. *Acta Med. Port*. **2022**. 35(5), 357–366. doi: 10.20344/amp.16574.
52. Fenizia, C.; Biasin, M.; Cetin, I.; Vergani, P.; Mileto, D.; Spinillo, A.; Gismondo, M.R.; Perotti, F.; Callegari, C.; Mancon, A.; et al. Analysis of SARS-CoV-2 vertical transmission during pregnancy. *Nat. Commun*. **2020**, 11, 5128. https://doi.org/10.1038/s41467-020-18933-4
53. Garcia-Ruiz, I.; Sulleiro, E.; Serrano, B.; Fernandez-Buhigas, I.; Rodriguez-Gomez, L.; Fernandez, D.S.-N.; Anton-Pagarolas, A.; Esperalba-Esquerra, J.; Frick, M.A.; Camba, F.; et al. Congenital infection of SARS-CoV-2 in live-born neonates: A population-based descriptive study. *Clin. Microbiol. Infect*. **2021**, 27, 1521.e1–1521.e5. https://doi.org/10.1016/j.cmi.2021.06.016.
